# Profiling Laboratory Biomarkers Associated with COVID-19 Disease Progression: A Single-Center Experience

**DOI:** 10.1155/2021/6643333

**Published:** 2021-02-09

**Authors:** Maria Khan, Noman Shah, Hina Mushtaq, Valeed Jehanzeb

**Affiliations:** Pathology Department, Rehman Medical Institute, Peshawar, Pakistan

## Abstract

**Background:**

There is clinical importance to investigate the disease progression through potential biomarkers of SARS-CoV-2 infection. In the present study, we aim to evaluate the significance of inflammatory markers in different categories of COVID-19 in admitted patients.

**Methods:**

In a single-center, observational study of 50 in-hospital patients who were diagnosed with COVID-19 and admitted to the intensive care unit of a tertiary care hospital in Peshawar, infection biomarkers, including hs-CRP, serum ferritin, serum creatinine, ALT, ALP, cardiac troponin-I, and IL-6 were analyzed.

**Results:**

The median age was 61.3 years. 70% (35) were male while 30% (15) were female. We noted significantly increased hs-CRP (9.32 mg/dL ± 10.03) and ferritin levels (982.3 ng/ml ± 601.9). A noteworthy increase was observed in neutrophil count (11.05 × 10^9^/L) and a decrease was observed in lymphocyte count (0.27 × 10^9/^L) (*P* < 0.05), and the platelet count was borderline decreased (244.1 × 10^9^/L). IL-6 levels were markedly increased in all admitted patients (100.2 pg/ml ± 122.2 pg/ml).

**Conclusion:**

The serum levels of CRP, troponin-I, ALP, ALT, serum creatinine, and ferritin are markedly increased in COVID-19 patients. Increased CRP and ferritin levels were also associated with secondary bacterial infection and poor clinical outcomes.

## 1. Introduction

SARS-CoV-2 belongs to the Coronaviridae family of enveloped, positive-sense single-stranded RNA group of viruses, mainly causing respiratory, gastrointestinal, hepatic, and neurological manifestations [1]. SARS-CoV-2 belongs to group 2B with around 70% resemblance to SARS-CoV-1 in nucleic acid sequence [2]. In the beginning, the laboratory findings depicted the cytopathic effect of SARS-CoV-2, leading to lung damage, apparent from pathological examination [3]. With disease progression, the lymphocyte count in the blood decreases drastically, along with an increase in the neutrophil count and a decrease in the platelet number, with prolonged activated thromboplastin time (APTT) and raised C-reactive protein, cardiac enzymes, and liver function tests. Inflammatory cytokine secretion (IL-1RA, IL-1B, IL-6, IL-7, and IL-8) is associated with cytokine storm and contributes to the pathogenesis of severe cases of COVID-19 [4]. Usually, IL-6 is secreted from monocytes and interstitial fibroblasts or alveolar macrophages of lung tissues [5, 6]. Disease severity, e.g., disseminated intravascular coagulation (DIC), acute respiratory distress syndrome (ARDS) in lungs, cardiovascular injuries (ischemia, pulmonary thromboembolism, and deep venous thrombosis), and cerebral infarctions (embolism) due to SARS-CoV-2 virus, cannot be anticipated with certainty using laboratory biomarkers for D-dimer, prothrombin time (PT), and activated partial thromboplastin time (aPTT) [7]. Largely, clinical and laboratory parameters contributing to complications were elderly, dyspnea, decreasing oxygen saturation, aspartate aminotransferase (AST), elevated neutrophil count, gamma-glutamyltransferase levels, and lactate dehydrogenase, raised CRP, high serum ferritin level, and elevated interleukin-6 [8]. Specific blood parameters, such as lymphopenia, and certain chemical features, in particular, troponin-I, serum creatinine, alanine aminotransferase, and alkaline phosphatase were delineated to be linked with the severity of COVID-19 [9]. Notably, increasing serum CRP levels corresponded to disease progression, serving as an early predictor for COVID-19 complication, prior to indications of critical findings with CT scan [10]. Serum ferritin rises proportionately in reaction to the inflammatory process, a well-known biomarker in a variety of diseases; additionally, the infamous biomarker of neutrophil-to-lymphocyte ratio (NLR) serves as a prognostic indicator for fatality in patients with different inflammatory manifestations [11]. Some studies have emphasized the role of high-sensitivity cardiac troponin-I (hs-TnI) being a contributing factor to poor prognosis [12]. With a crude mortality rate of about 2.3% [13], therefore, the prompt spread of disease requires instant labeling of cases into risk group categories following laboratory-confirmed diagnosis, to make sure optimum resource provision and implement swift management protocols [14]. The biomarkers we review in this study are serum ferritin, IL-6, hs-CRP, white cell count (WCC), platelet count, aPTT, alkaline phosphatase (ALP), alanine aminotransferase (ALT), cardiac troponin-I, and renal markers. This study aimed at investigating the correlation of different laboratory inflammatory biomarkers among patients with COVID-19.

## 2. Methodology

The study was approved by the Institutional Ethical Board of Rehman Medical Institute, Peshawar. Total patients admitted to Rehman Medical Institute (Peshawar, Pakistan) between 17 June 2020 and 2 July 2020 with laboratory-confirmed SARS-CoV-2 infection were *n* = 87, among which 50 were included where the other 37 patients had either mild symptoms or asymptomatic admitted for some other noninfectious ailments. Demographic and epidemiological statistics, such as age, sex, and disease history, were gathered upon admittance. For laboratory confirmation, real-time reverse transcriptase-polymerase chain reaction (RT-PCR) was used as gold standard, according to the recommended protocol of the hospital. All baseline serum samples were collected immediately after admission for total blood count, ALT, ALP, CRP, aPTT, IL-6, and ferritin. For quality control, serum samples from 25 healthy volunteers were collected.

Patients were categorized into moderate, severe, and critical COVID-19 groups according to China's Novel Coronavirus Pneumonia Diagnosis and Treatment Guideline (5th edition) [15, 16].

### 2.1. Moderate Group

Moderate group included patients with fever, respiratory manifestations, and imaging findings of pneumonia.

### 2.2. Severe Group

Severe group included patients with the following additional findings: respiratory distress, RR ≥30 times/minute; oxygen saturation (SpO_2_) ≤93%; oxygen partial pressure (PaO_2_)/oxygen concentration (FiO_2_) in arterial blood ≤300 mmHg; and >50% lung imaging deteriorating within 24–48 h.

### 2.3. Critical Group

Critical group included patients having any of the following findings: respiratory failure and mechanical ventilation essential; shock; multiorgan failure; and need for intensive care unit.

### 2.4. Laboratory Investigation

Roughly 2∼4 ml of peripheral blood was collected from subjects of the categorized groups, and serum was separated by 2000 rpm/20 min centrifugation. Serum cytokines were analyzed using the Elecsys IL-6 immunoassay and electrochemiluminescence immunoassay (ECLIA) performed on a COBAS e411 analyzer (Roche Ltd.). It is an in vitro diagnostic test for the quantitative determination of IL-6 in human blood. 25 *μ*l of serum was incubated with biotinylated monoclonal IL-6 antibody, labeled with a ruthenium complex and streptavidin-coated microparticles, forming a sandwich complex with the antigens present in the sample, and the resulting chemiluminescence is then measured by using photomultiplier. Data were statistically analyzed by SPSS for Windows 25.0 (SPSS Inc., Chicago, IL). A value of *p* < 0.05 was considered statistically significant.

## 3. Results

Among the total of 50 admitted patients, 30% were classified as Critical group (*n* = 15), 48% were classified as Severe group (*n* = 24), and 22% (*n* = 11) were classified as Moderate group. The mean age of patients was 61.3 years (SD = 14.4). 70% (35) were male, while 30% (15) were female.  Table 1 shows the mean value of biomarkers in total patients.

Duration for hospital admission to intubation period variegated from less than two hours to 9 days (median 4 days). Elevated interleukin-6 (IL-6), troponin-I, and ferritin levels were influentially accompanied by mechanical ventilation need. In total, 38/50 (68%) patients deteriorated during hospitalization and required mechanical ventilation. The mortality rate was 92% among patients on mechanical ventilation.  Table 2 shows the individual biomarker profile of each patient.

Among the hematologic parameters, total hemoglobin levels were low in 16% (*n* = 8) of patients, total platelet count was low in 18% (*n* = 9) of patients, total leukocyte count was high in 44% of patients, neutrophil count was high in 94% (*n* = 47) of patients, lymphocyte count was low in almost 50% (*n* = 25) of patients, whereas neutrophil-to-lymphocyte ratio was increased in 92% (*n* = 46) of patients admitted.

Cardiac markers, hs-troponin-I levels, were high in 72.5% (*n* = 36), serum ferritin values were high in 96% (*n* = 48), serum alkaline phosphatase was high in 36% (*n* = 18), serum creatinine was high in 60% (*n* = 30), CRP was high in 82% (*n* = 41), and serum ALT was raised in 36% (18) of patients.

Similarly, serum ferritin levels directly correlated with increasing levels of serum ALP (*r* = 0.548, *P* < 0.0001), serum troponin (*r* = 0.37, *P* < 0.05), CRP (*r* = 0.306, *P* < 0.05), and neutrophil count (*r* = 0.79, *P* < 0.0001). Serum alkaline phosphatase positively correlated with levels of CRP values (*r* = 0.439, *P* < 0.05) and neutrophil count (*r* = 0.47, *P* < 0.05). There was a positive correlation of aPTT with neutrophil-to-lymphocyte ratio (*r* = 0.45, *P* < 0.005), TLC (*r* = 0.363, *P* < 0.05), and hs-CRP (*r* = 0.339, *P* < 0.05). In contrast, negative correlation of platelet count with IL-6, serum creatinine, ALP, hs-troponin-I, TLC, serum ALT, and serum CRP was noted. Lymphocyte count also negatively correlated with IL-6, serum ferritin, ALP, CRP, aPTT values, serum creatinine, and hemoglobin levels.

## 4. Discussion

The documentation of prognostic variables may contribute to the evidence-based management of patients with COVID-19. In this study, we evaluated the demographic, diagnostic, and clinical characteristics of 50 patients with COVID-19 admitted to ICU and determined probable inflammatory markers affecting the clinical outcomes of patients. It should be noted that biomarkers are used in the diagnosis of various clinical conditions reflecting pathological progression. We established ALT  elevation in 36% of cases, CRP was raised in 82%, alkaline phosphatase was raised in 36%, and 96% of patients had markedly increased serum ferritin levels. Moreover, in severe and critical categories, IL-6 levels were markedly raised. Particularly, this study indicated inflammatory events due to cytokine storms, high CRP, and markedly raised serum ferritin levels among critically ill patients contributing to worsened disease state, clearly highlighting the management of lung damage as a crucial step. Apart from this, our findings suggest that the expression of IL-6 and CRP accounts for timely diagnosis of patients having severe disease, keeping in view the substantial burden of healthcare in individual hospitals. Subsequently, hyperferritinemic cases were among the male and elderly age group, correlating towards more severe disease than those with normal serum ferritin levels. Besides, these particular patients had considerably raised serum creatinine, ALP, and ALT, decreased lymphocyte count, and increased levels of inflammatory biomarkers, such as serum CRP [17]. The association of hyperferritinemia and complications in patients with SARS-CoV-2 is indistinguishable, but the contributing factors such as IL-6 directly affecting ferritin synthesis can be a possibility [18, 19]. Similarly, ferritin functions as iron binding and storage and is indirectly associated with immunity and inflammatory responses of the body [20]. The link between CRP and COVID-19 has been emphasized by a study at a tertiary care hospital in Wuhan, where most of the patients in the severe category exhibited increased parameters in comparison with the nonsevere group (57.9–33.2 mg/L) [21]. Similarly, another study established disease progression to a severe state due to CRP levels of >41.8 mg/L [22]. In critically ill patients, persistently raised IL-6 levels released by activated macrophages correlate directly to high viral RNA load which in turn leads to ARDS deterioration [23–25]. Comparatively, platelet count was significantly decreased in critically ill patients and nonsurvivor than survivors [26]. Yang et al. in one of his studies reported lymphopenia among 80% of critically ill patients [6], while Chen et al. stated lymphopenia in 25% of patients having mild infection [24]. However, in the present study, 50% of patients suffered from lymphopenia. Interestingly, the levels of other white cell counts, monocytes, eosinophils, and basophils, also decreased, ensuing greater neutrophil-to-lymphocyte ratio that portrays poor prognosis [27]. In our study, the mean NLR was 15.6 and was significantly raised in 92% of patients. A meta-analytic study revealed significantly lower platelet counts in 1799 patients with severe COVID-19 infections [28].

In addition, cardiac enzymes and muscular biomarkers were also raised in severe and critical COVID-19 patients. Another prospective study of 179 patients with COVID-19 reported cardiac troponin-I ≥0.05 ng/mL, amongst high risk factors and predictors of mortality [29]. Similarly, we found 72.5% of patients having elevated hs-troponin-I, with a mean value of 2972.3 ng/dl. In the same way, 416 hospitalized patients of COVID-19 reported high-sensitivity troponin-I elevation among 1 in 5 patients on admission [30]. Timely recognition of cardiac injury by deranged hs-TnI levels supports incorrect triage measures and directs towards the usage of vasopressors and inotropes. Significantly raised levels of serum urea and creatinine also contribute to disease severity [31]. In a wide retrospective multicenter study involving 5771 patients with COVID-19, Lei et al. reported a strong association of serum ALT levels with mortality risk compared to other liver injury parameters [32]. In the present study, 36% of patients had elevated serum ALT levels, with a mean of 48.94 U/L.

## 5. Conclusion

From the time when the pandemic crisis originated, it is of utmost importance to analyze the clinical implication of hematologic, biochemical, inflammatory, and immunologic biomarkers in patients with or without severe/fatal forms of COVID-19. Inference gathered till date suggests the strong clinical correlation among varied levels of inflammatory markers that confer to the severity of COVID-19, alternatively being used as an adjunct in guiding towards treatment options and criteria for admission, thereby improving prognosis and minimizing mortality rate. Our findings indicated that C-reactive protein level, serum ferritin, and troponin-I were autonomous predictors of severity in COVID-19 cases. Independently, lymphocyte count has been recommended as a probable biomarker of the disease, showing consistently low levels reported in critically ill patients. Elevated serum CRP as well as ferritin levels might have a clinical correlation with secondary bacterial infection, contributing to poor prognosis.

## Figures and Tables

**Table 1:** Mean value of inflammatory biomarkers.

Variable	Mean	Standard deviation
Serum interleukin-6	100.20 pg/mL	122.2 pg/mL
Serum ferritin	982.3 ng/ml	601.9 ng/ml
Serum alkaline phosphatase (ALP)	109.2 U/L	41.1 U/L
Ultrasensitivity cardiac troponin-I	2972.3 ng/dL	7615.8 ng/dL
Serum creatinine	2.09 mg/dL	1.80 mg/dL
Serum C-reactive protein (CRP)	9.32 mg/dL	10.03 mg/dL
Serum alanine aminotransferase (ALT)	48.94 U/L	38.49 U/L
Activated partial thromboplastin time (APTT)	31.76 seconds	21.52 seconds
Hemoglobin levels (Hb)	12.54 g/dL	2.2 g/dL
Total leukocyte count (TLC)	12.97 × 10^6^/*µL*	8.04 × 10^6^/*µL*
Platelet count	244.1 × 10^9^/L	121.72 × 10^9^/L
Neutrophil count	11.0502 × 10^9^/L	2.76044 × 10^9^/L
Lymphocyte count	0.2754 × 10^9^/L	1.02173 × 10^9^/L
Neutrophil-to-lymphocyte ratio (NLR)	15.6	16.50

**Table 2:** Patients' biomarker profile and blood counts (*n* = 50).

S. no.	Age (years)	IL-6 levels (<7 pg/ml)	Ferritin levels (17–230 ng/ml)	APTT (28 seconds)	Creatinine levels (0.7–1.2 mg/dl)	ALP levels (39–117 U/L)	Troponin levels (0.33 ng/dl)	CRP levels (<0.5 mg/dl)	ALT (10–50 U/L)	Hb (12.5–16.5 g/dl)	Total leukocyte count (4–11 × 10^6^/*μ*l)	Platelet count (150–450 × 10^9^/L)	Lymphocyte count (1.5–4 × 10^9^/L)	Neutrophil count (2–7.5 × 10^9^/L)
1	71	98	634	38	3.5	95	1161	8.3	78	11.1	7.75	245	1.39	9.2
2	64	77	316	33	0.9	86	603	5.6	45	12.2	12.6	164	1.4	11.4
3	57	44	288	30	1.1	44	29	6.2	67	14.2	5.6	139	2.32	12.7
4	41	122	1675.6	45.4	9.5	175	7816	9.21	69	7.7	10.4	223	2.32	15.8
5	88	65	461.54	36.3	1.1	103	532	15.89	10	10	15.44	102	2.71	10.7
6	61	149	1147.69	20.6	1.4	109	7637	1.76	30	13.9	28.23	219	0.5	12.2
7	51	29	1206	28.7	1.2	108	7637	25.11	31	13.7	12.47	179	1.45	11.85
8	61	68.9	1978	30.5	2.3	126	7759	28.5	59	10.3	14.2	167	1.3	16.5
9	57	40.5	503	26	0.6	100	112	2.43	37	15.1	6.89	224	1.22	14.8
10	55	121	1675	48	1.8	154	6702	0.25	137	14.4	10.7	586	0.37	12.6
11	74	82	429.07	28	0.9	98	1179	0.67	18	11.6	9.32	317	0.26	11.2
12	66	61.7	427.31	30.7	1.4	81	99	0.9	11	11.6	9.32	317	0.26	8.7
13	56	88.6	819	31	1.4	110	4107	5.6	22	13.4	8.11	115	2.71	9.12
14	71	92.3	1247.8	33.8	1.1	122	7985	0.21	136	13.8	27.06	450	6.03	13.54
15	31	55.1	510.67	23.2	3.9	104	7.3	0.65	23	15.4	16.9	273	0.23	7.9
16	43	118	1847	22.2	5.2	116	81	0.84	39	15.5	14.2	344	1.56	8.8
17	54	59.7	312	28	0.6	101	5.4	1.81	59	12	11.7	409	1.78	7.6
18	57	143.7	519	120	3.8	115	25.9	28.3	11	9.6	37.78	209	0.62	8.4
19	58	29.3	234.03	30.1	08	166	11.9	3.27	15	11.7	5.98	288	1.37	7.8
20	86	115.9	412	28	0.9	99	2635	11	52	15.2	4.77	124	0.9	9.6
21	49	133	647	23.8	0.6	52	0.02	0.08	18	13.6	9.6	290	0.67	10.2
22	37	19	93.4	28	0.7	48	0.2	0.09	72	11	11.9	329	2.78	8.5
23	61	146	1687.1	21.2	2.4	186	33	32	83	12.2	12.92	370	1.46	14.7
24	56	89.2	726	27	2.8	120	335	0.23	40	-	11.9	218	0.6	11.5
25	53	109	1192.6	28.6	1.3	154	616	4.91	77	18.6	6.3	152	1.16	15.3
26	71	44.7	315	28	1.3	97	704	18.5	72	12.2	30.5	164	0.44	9.1
27	76	119.5	629	23.1	1.4	145	6402	8.2	39	7.6	4.3	23	1.93	10.6
28	51	128.4	2000	10	1	91	762.4	0.02	38	13.5	6.72	495	2.2	10.1
29	86	107	1546	17	1.4	112	5018	11.8	45	12.5	7.03	154	0.91	12.9
30	66	76.1	1675	128	2.7	192	37.8	29.57	28	11.6	19.01	193	0.13	13.5
31	81	55	958	28	2.7	155	6275	1.61	41	11.3	28.79	414	0.64	8.3
32	21	26	520	28	2.1	76	0.41	1.61	41	11.3	28.79	414	0.64	7.8
33	61	95	1834	24.6	1	85	17229	15.48	13	12.9	6.6	207	1.95	15.1
34	56	189	1944	14.1	0.8	113	50000	24.11	22	16.2	10.76	235	0.86	15.9
35	66	12	312	28	1.1	81	0.25	3.27	51	10.3	10.8	124	3.05	6.6
36	77	37	1482	23	3.3	149	35.6	8.62	66	13.8	15.3	186	0.8	11.4
37	71	111	1158	26.9	2.6	44	444	11.98	24	10.1	6.94	143	0.7	9.7
38	71	59.6	1603	18.5	5	158	561	19.21	60	8.12	8.65	123	0.5	12.6
39	61	25.9	609	28	0.7	40	35.7	12.50	99	12.5	6.05	277	2.18	10.9
40	68	114	1675	25.5	1.2	160	277.6	33.78	227	11.2	30.1	425	0.59	13.3
41	26	120.8	1703	24.5	0.6	186	2.63	0.27	18	11.9	6.75	157	1.39	14.4
42	85	135	2000	18	2.2	140	889.5	0.27	18	11.9	6.75	157	1.39	15.2
43	64	118	1809	23	4.8	131	203	5.69	13	12	3.39	210	0.62	15
44	62	53.1	585.3	27.2	1.4	62	117	0.14	46	15	19.69	442	1.08	8.9
45	71	66	723.5	26.4	1.9	71	319	1.87	38	11.9	10.79	230	0.74	9.5
46	58	19	222.9	19	1.1	32	2.6	3.24	29	16.2	18.41	232	0.15	7.5
47	67	80.4	938.1	27	1.8	109	3.4	27.33	66	12.6	8.56	168	0.8	8.8
48	57	69.3	551	28	0.9	54	10.4	15.5	30	13.9	9.35	174	0.9	7.4
49	74	92.1	748.4	80.6	1.9	67	71.8	9.56	47	11.1	13.6	386	1.36	9.3
50	64	899	588.9	23.5	1.6	142	2105	8.53	37	15.1	9.16	23	0.45	8.1

## Data Availability

The data used to support the findings of this study are included within the article.

## Abbreviations

aPTT: Activated partial thromboplastin time
ARDS: Acute respiratory distress syndrome
ALT: Alanine aminotransferase
ALP: Alkaline phosphatase
CDC: Centers for Disease Control and Prevention
COVID-19: 2019 novel coronavirus disease
DIC: Disseminated intravascular coagulation
hs-CRP: High-sensitivity C-reactive protein
ICU: Intensive care unit
IL-6: Interleukin-6
NLR: Neutrophil-to-lymphocyte ratio
PO_2_: Partial pressure of oxygen
PT: Prothrombin time
RNA: Ribonucleic acid
SpO_2_: Oxygen saturation
SARS-CoV-2: Severe acute respiratory syndrome coronavirus 2
TLC: Total leukocyte count.
